# Reproductive Strategies in Mediterranean Legumes: Trade-Offs between Phenology, Seed Size and Vigor within and between Wild and Domesticated *Lupinus* Species Collected along Aridity Gradients

**DOI:** 10.3389/fpls.2017.00548

**Published:** 2017-04-13

**Authors:** Jens D. Berger, Damber Shrestha, Christiane Ludwig

**Affiliations:** ^1^CSIRO Agriculture and FoodWembley WA, Australia; ^2^Centre for Legumes in Mediterranean Agriculture, Faculty of Natural and Agricultural Sciences, University of Western AustraliaCrawley, WA, Australia

**Keywords:** adaptation, crop evolution, terminal drought, phenology, seed size, early vigor, hard seed breakdown

## Abstract

To investigate wild and domesticated Mediterranean annual reproductive strategies, common garden comparisons of Old World lupins collected along aridity gradients were initiated. These are excellent candidates for ecophysiology, being widely distributed across contrasting environments, having distinct domestication histories, from ancient *Lupinus albus* to recently domesticated *Lupinus angustifolius* and *Lupinus luteus*, facilitating the study of both natural and human selection. Strong trade-offs between seed size, early vigor and phenology were observed: vigor increasing, and flowering becoming earlier with increasing seed size. Despite large specific differences in all these traits, natural and human selection have operated in very similar ways in all 3 species. In wild material, as collection environments became drier and hotter, phenology became earlier, while seed size, early vigor and reproductive investment increased. Wild and domesticated germplasm separated along similar lines. Within similar habitats, domesticated material was consistently earlier, with larger seeds, greater early vigor and higher reproductive investment than wild, suggesting selection for both early establishment and timely maturity/drought escape in both domesticated and wild low rainfall ecotypes. Species differences reflected their distribution. Small and soft-seeded, low vigor *L. luteus* had a late, rainfall-responsive phenology specifically adapted to long season environments, and a narrow coastal distribution. *L. angustifolius* was much more conservative; more hard-seeded, flowering and maturing much earlier, with a wide Mediterranean distribution. *L. albus* flowered earlier but matured much later, with longer reproductive phases supporting much larger seed sizes and early vigor than either *L. luteus* or *L. angustifolius*. This ruderal/competitive combination appears to give *L. albus* a broad adaptive capacity, reflected in its relatively wider Mediterranean/North African distribution.

## Introduction

Despite their high value and rotational benefits, cool season legume crops can struggle to retain their place in farming systems because of their perceived riskiness (Pannell, 1995). There is a wide range of biotic (weeds, pests and diseases) and abiotic stresses, such as drought, heat and cold that impact grain legume production. Understanding species' adaptive responses to such stresses is the key to genetic risk mitigation, by ensuring that crops with an appropriate assemblage of adaptive traits are grown in fitting production niches. To define species adaptive potential it is important to work with a broad range of genetically diverse material, rather than the narrow band of elite cultivars that typifies many modern grain legume crops (Abbo et al., 2003; Berger et al., 2013). Genetic resource collections that sample a wide range of habitats are an ideal vehicle for this.

Here we focus on adaptive strategies among the “Old World” lupins, (*Lupinus albus* L., *L. angustifolius* L., *L. luteus* L.) widely collected along terminal drought stress gradients across the Mediterranean basin (Berger et al., 2008). The Mediterranean climate poses wide ranging challenges to plant adaptation. Despite the almost ubiquitous cool, wet winters and hot, dry summers there is wide spatial and temporal variation. Seasonal rainfall, temperature, relative humidity, solar radiation, and wind speed vary across the Mediterranean basin (Hijmans et al., 2005), coupled with differences in aspect, slope, soil depth, pH, fertility, water holding capacity, land management and grazing intensity. As a result of this manifold variation, broadly distributed species typically encounter contrasting habitats. These exert differential selection pressure, leading to the formation of distinct specifically adapted ecotypes. Understanding the extent to which the “Old World” lupins form distinct ecotypes will help breeders to maximize productivity in their target environments, and address gaps-such as the current lack of a long season narrow–leafed lupin cultivar (Berger et al., 2012a; Chen et al., 2016).

A range of reproductive strategies has been invoked to explain specific adaptation in Mediterranean winter annuals, typically emphasizing phenology and its trade-offs against other plant attributes. Grime's (1979) triangle balances phenology against biomass production/resource acquisition along the disturbance/competition continuum. In the Mediterranean context, Grime's (1979) triangle predicts that conservative ruderal strategies (rapid growth rates/short life cycles) are favored in environments “disturbed” by terminal drought: high temperatures, and low, uncertain rainfall. Conversely, cooler, longer season environments with high, consistent rainfall are more likely to select for longer lifecycles supporting the development of competitive traits that facilitate resource capture, such as high investment in above and below ground biomass, ultimately leading to greater seed production. Grime's (1979) predictions are well supported among Mediterranean annuals, with a strong focus on pasture legumes (see references in Berger and Ludwig, 2014). This conservative-competitive trade-off has far reaching ramifications. In *L. luteus*, in addition to phenology/productivity trade-offs, rates of water-use, timing of stress onset and drought tolerance capacity are all traded-off among low and high rainfall ecotypes (Berger and Ludwig, 2014). Indeed, we suggest that the expression of drought escape, postponement and tolerance is closely linked to the phenology-productivity trade-off, producing integrated adaptive strategies that suit specific environmental niches (Berger et al., 2016) that should be exploited by farmers.

Dormancy, dispersal and seed size are also traded off in adaptation. Dormancy and dispersal facilitate escape from stress in space and time, while larger seeds increase the probability of successful establishment under unfavorable conditions (Venable and Brown, 1988). Accordingly, seed size is predicted to increase as dormancy or dispersal decrease (Venable and Brown, 1988), while the latter are also negatively correlated, since having more of one reduces the need for the other (Rubio de Casas et al., 2015). Germination behavior has been divided into 2 categories (Gremer et al., 2016). Predictive or plastic germination that responds to cues signaling the onset of favorable conditions for seedling growth are expected to be important in predictable climates, and habitats where strong competitive pressure selects for early emergence to acquire resources before they are lost. Alternatively, bet-hedging germination, where cohorts of seed germinate at different times spreads risk in time. Diversified bet-hedging trades off reduced fitness at any given time point (because only a fraction of the population is able to germinate) against reduced variance in fitness over time, and is only adaptive when environmental conditions vary from generation to the next (Donaldson-Matasci et al., 2013).

In Mediterranean annual legumes physical dormancy (also referred to as hardseededness), caused by a water-impermeable seed coat that tends to breaks down during fluctuating summer temperatures, is the most important mechanism preventing inopportune germination (Norman et al., 2002), protecting populations against false breaks before the onset of the rainy season. If there is variability in the degree of physical dormancy loss this mechanism may also play a diversified bet-hedging role. Therefore, given that rainfall variability tends to increase with aridity, hard seed incidence may increase in low rainfall environments (Hacker, 1984; Hacker and Ratcliff, 1989; Piano et al., 1996). Alternatively, in-season phenology may itself be traded-off against hardseededness (Norman et al., 2005). Genotypes with a high proportion of hard seed and low rate of dormancy loss can afford a more competitive, later phenology than those with soft, permeable seeds, if their additional productivity during favorable years establishes a larger seedbank than those of earlier, more ruderal genotypes. Phenology may also be traded-off against seed size because smaller seeds fill more quickly, allowing small seeded species to run a later phenology than large seeded species from the same environment (e.g., small seeded *Trifolium clusii* and *T. glanduliferum* vs. large seeded *T. purpureum* in Northern Israel) (Cocks, 1999). Finally, increasing seed size and its associated competitive advantages are traded off against fecundity (Venable and Brown, 1988; see also references in Norman et al., 2005).

Clearly there are a wide range of strategies that allow annual plants to adapt to Mediterranean climates. While studies within and between widely distributed species are most informative (Thompson, 2005), the study of annual species has often focused on a narrow germplasm and geographic range (see references in Berger and Ludwig, 2014). The “Old World” lupin species of the present study are interesting because they are large seeded, and adapted to neutral to acid, low water-holding capacity soils. Consequently seed size trade-offs are investigated at a different scale than the smaller seeded pasture legumes of previous studies (Cocks, 1999; Norman et al., 2005). Furthermore, phenology/productivity trade-offs may be stronger than in species from higher water holding capacity soils that buffer the onset of terminal drought to some extent. These include many of the species listed in Berger and Ludwig (2014), such as *Triticum dicoccoides* (Kato et al., 1998), *Hordeum spontaneum* (Volis, 2007), *Medicago* (Yousfi et al., 2010), and *Trifolium* species (Norman et al., 2002), typically collected from finer-textured sand- or clay-loams Moreover, these “Old World” lupins have contrasting distributions, seed sizes, and domestication histories. *L. albus* is a very widely distributed Bronze Age domesticate with large to very large seeds grown on mildly alkaline to acidic sands and loams. Conversely, the smaller seeded *L. luteus* and *L. angustifolius*, domesticated in the last 200 years, have limited to intermediate natural Mediterranean distributions on acid, sandy soils (Gladstones, 1974; Hondelmann, 1984; Huyghe, 1997; Zohary and Hopf, 2000).

Given these differences, we were interested to discover the extent to which these Old World lupins trade-off phenology, productivity, seed size and hardseededness along collection site terminal drought stress gradients. Is there a single, common reproductive strategy within the genus for coping with the transition from low to high rainfall environments, or are there specific differences which relate to seed size, distribution or domestication history? Beyond the accepted “domestication syndrome” traits (Meyer et al., 2012), how do wild and domesticated lupins differ, and did ancient and modern domestication select for similar or different reproductive strategies?

## Materials and methods

### Germplasm selection and habitat characterization

**Figure 1:**
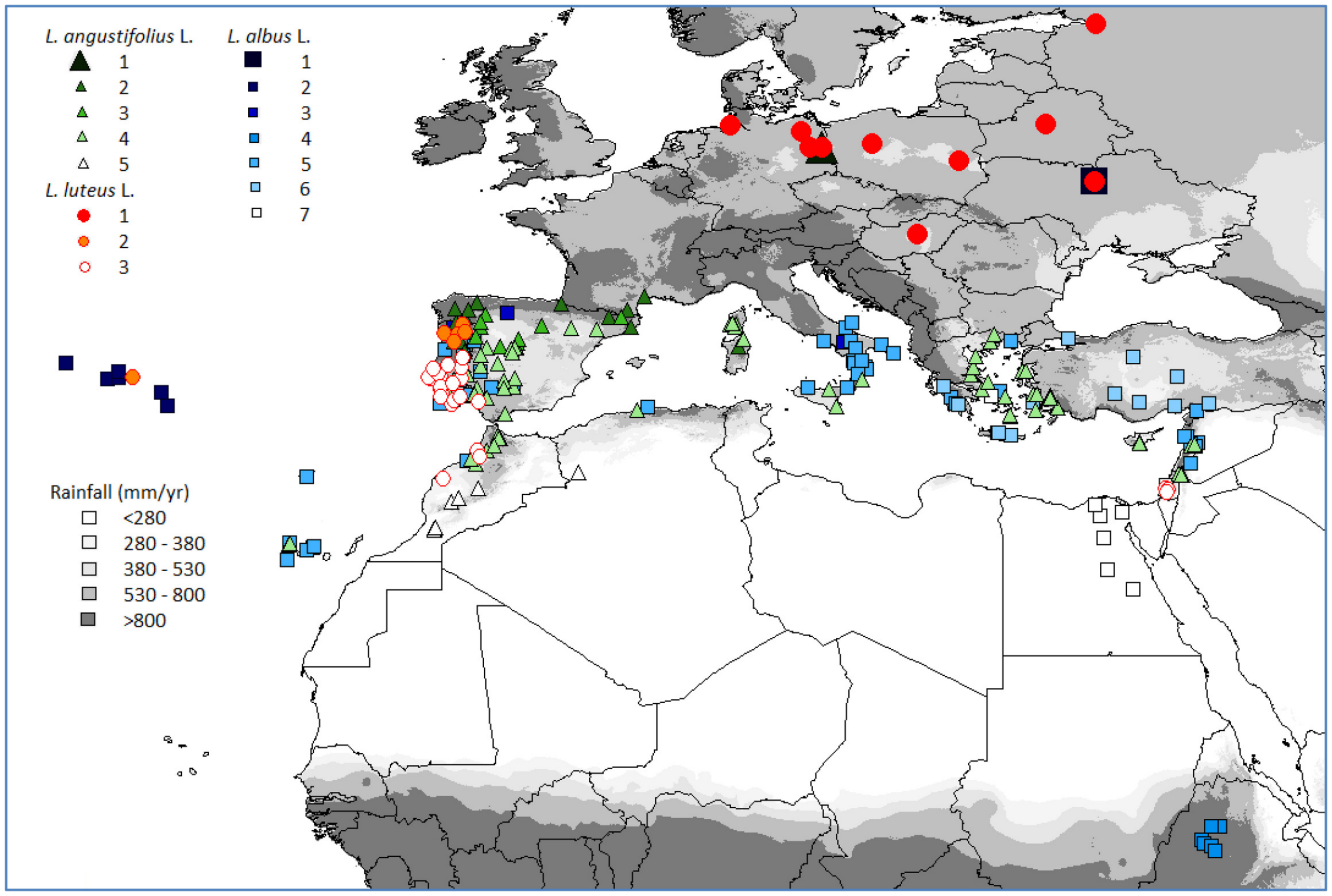
**Northern hemisphere collection sites of old world lupin genotypes evaluated in the present study, classified by species and habitat cluster (see Table 1)**.

All experiments in the present study were based on the evaluation of wild and domesticated old world lupins (*L. albus, L. angustifolius* and *L. luteus*) collected from a wide range of habitats representing each species' distribution range (Figure 1, Table 1; Berger et al., 2008). Collection site climate was characterized in Berger et al. (2008) by defining typical growing season phenology for each site and calculating bioclimatic variables for vegetative and reproductive phases based on long term average precipitation (monthly totals and coefficients of variation), temperature, daylength, relative humidity, sunshine percentage, wind speed, and precipitation (Hijmans et al., 2005). Relationships among these bioclimatic variables, as well as latitude, longitude and altitude were simplified using principal components analysis (PCA) performed within each species. The resultant scores for PC 1–4, capturing 78.2–90.6% of variance, were used to classify collection sites by hierarchical clustering (Ward's method, SPSS Version 10).

**Table 1:** **Provenance and seasonal collection site climate (details in Berger et al., 2008) of old world lupin common garden field experiments at in 2007, 2008 and 2015 (hard seed breakdown)**.

**^a^Clust**	**Description**	**Germplasm *n***			**Rainfall (mm)**			**Rain days**		**Frost days**	**Mean temp (°C)**		**Temp change (°C/day)**	**Summer temp (°C)**		**^b^Status**			**Origin**
		**07**	**08**	**15**	**Pre-seas**	**Veg**	**Rep**	**Veg**	**Rep**	**Veg**	**Veg**	**Rep**	**Rep**	**Max**	**Range**	**Br**	**Lr**	**W**	
^c^***L. albus*** **L**.		88	17													14	58	16	
1	European spring-sown: long daylength, rapidly warming, cool veg phase, med rainfall, low terminal drought.	1			310	102	226	24	42	7	12.0	18.9	0.01	24.7	11.4	1			UKR, 1
2	Mediterranean (Iberian) long season: cool temp, high, frequent rainfall, no terminal drought.	6	2		104	515	521	69	96	4	14.5	15.8	0.05	23.8	9.4		6		PRT, 6
3	Mediterranean long season: low temp (cold, frosty veg phase), high, frequent rainfall, warming rep phase; low terminal drought.	5			54	581	234	81	42	56	5.8	15.0	0.09	23.6	12.5		2	3	ITA, 3; ESP, 1; PRT, 1
4	Ethiopian highlands: warm, wet veg phase, cool, low rainfall rep phase; low terminal drought.	6	3		773	668	61	33	9	2	16.2	16.3	0.01	27.0	16.3		6		ETH, 6
5	Average Mediterranean climates: intermediate terminal drought.	48	6		41	376	234	47	41	18	12.2	16.3	0.09	29.2	13.3		36	12	ITA, 15; ESP, 11; PRT, 8; GRC, 6; SYR, 6; MAR, 1; DZA, 1
6	Mediterranean spring-sown: low rainfall, warm rep phase with rapid temp increase; high terminal drought.	14	3		552	91	13	19	4	2	16.4	23.6	0.10	30.0	14.0		14		TUR, 7; GRC, 7
7	Southern Mediterranean: low rainfall, warm rep phase with rapid temp increase; high terminal drought.	8	3		5	47	32	8	10	3	16.6	18.2	0.12	34.6	14.8		8		EGY, 6; ISR, 1; GRC, 1
***L. angustifolius*** **L**.		135	42	21												16	0	115	
1	European spring-sown: long daylength, rapidly warming, cool veg phase, med rainfall, low terminal drought.	1	3		269	91	186	26	40	11	11.1	17.9	0.02	23.8	12.1	1			DEU, 1
2	Mediterranean long season: high, frequent rainfall, cool veg phase; low terminal drought.	18	8	8	64	558	324	76	62	50	8.3	15.6	0.09	26.4	13.8			18	ESP, 10; PRT, 4; FRA, 3; ITA, 1
3	Mediterranean (Iberian) long season (cool): intermediate, frequent rainfall, cold frosty veg phase; med terminal drought.	13	1		50	391	164	85	39	97	5.7	16.0	0.10	26.8	16.7			11	ESP, 11
4	Average Mediterranean climates: intermediate terminal drought.	92	23	9	46	345	226	44	38	16	12.2	15.9	0.10	31.2	15.6	15		77	ESP, 20; AUS, 19; GRC, 17 ; MAR, 10; ISR, 5; FRA, 4; ITA, 4; PRT, 3; SYR, 3; TUR, 3; CYP, 2; DZA, 1; ZAF, 1
5	Nth Africa: low, infrequent rainfall, high sun hours, rainfall variability: high terminal drought.	7	5	4	26	145	135	18	20	21	11.4	16.3	0.10	33.7	19.6			7	MAR, 6; DZA, 1
	Unclassified	4	2													2		2	GRC, 2; CHL, 2
***L. luteus*** **L**.		71	29	13												35	0	36	
1	European spring-sown: long daylength, rapidly warming, cool veg phase, med rainfall, low terminal drought.	30	11	1	296	99	211	26	41	11	10.9	17.7	0.01	23.6	11.7	30			DEU, 6; BYS, 5; UKR, 5; HUN, 4; POL, 3; AUS, 2; RUS, 2; SUN, 1; LVA, 1; NLD, 1
2	Mediterranean (Iberian) long season: high, frequent rainfall, cool veg phase; low terminal drought.	7	7	6	70	645	416	76	72	27	10.5	15.5	0.08	26.2	13.2			7	PRT, 6; ESP, 1
3	Average Mediterranean climates: intermediate terminal drought.	32	11	6	47	368	224	45	40	4	13.5	16.2	0.08	30.1	15.2	5		29	PRT, 17; AUS, 9; MAR, 3; ISR, 3; ESP, 1; ZAF, 1
	Unclassified	2																	PRT, 2
**COMMON GARDEN FIELD TRIAL CLIMATE**																			
	2007 field trial				230	262	69	46	21	0	14.1	17.1	0.11						
	2008 field trial				258	187	101	32	36	0	12.5	16.3	0.07						

a *Cluster: collection sites were clustered largely on the basis of seasonal climate and described in Berger et al. (2008)*.

b *Status: Br, breeding material or released cultivar; Lr, landrace; W, wild germplasm. Numbers refer to 2007 evaluation*.

c *L. albus L. collection site climates above were re-analysed in light of the fact that landraces from the Balkans to Asia Minor are spring-(Mülayim et al., 2002; Mihailovic et al., 2008), rather than autumn-sown as assumed in Berger et al. (2008)*.

*Note that 2007 and 2008 field trial climate is also included to put these evaluations into context alongside germplasm collection site climate. Summer maxima and average temperature range are included as background for the physical dormancy work*.

These Ward's clusters formed the basis of the comparisons made in the present study. All 3 species were distributed along terminal drought stress gradients defined by reproductive phase rainfall and temperature; stress increasing with cluster number (Table 1). Thus, Cluster 1 (spring-sown Central Europe) had the lowest terminal drought stress, increasing through a range of Mediterranean regions, typically culminating in rapidly warming, low rainfall reproductive phase northern African environments (e.g., *L. angustifolius*, Cluster 5; *L. albus*, Cluster 7). *L. albus* was particularly widely distributed, from the Azores and Canary islands, through the Mediterranean basin, including the Nile valley and Ethiopian highlands (Figure 1). *L. albus* was largely domesticated (Table 1), with many Mediterranean, but also Ethiopian landraces (Cluster 3), reflecting its long history as a domesticated crop relative to other Old World lupins (Zohary and Hopf, 2000). Conversely, *L. luteus* and especially *L. angustifolius* were dominated by wild Mediterranean germplasm, but also included some cultivars from Europe and Mediterranean climates in Western Australia and South Africa. *L. luteus* was distributed less widely than the others, along a narrower terminal drought stress gradient, because even the most stressful habitat (Cluster 3) has relatively high reproductive phase rainfall and only a moderate temperature increase compared to *L. angustifolius* and *L. albus* (Table 1).

### 2007 Field evaluation

*Lupinus albus* (*n* = 88), *Lupinus angustifolius* (*n* = 133), and *Lupinus luteus* (*n* = 73) were acquired from the Australian Lupin Collection and evaluated as spaced plants in a common garden field experiment (RCBD, *n* = 3) in a sandy loam at CSIRO Floreat (−31.95 N, 115.79 E). Plots were hand planted on 9th July 2007 after vernalization to simulate an endemic Mediterranean environment, where lupins are vernalized by low temperatures early in their lifecycle. Seeds were scarified and allowed to swell at room temperature overnight, and then placed into moist petri dishes in dark growth cabinets for vernalization at 8°C for 16 days, using P-Pickle-T® prophylaxis against fungal infection. Seeds were inoculated with Nodulaid 100® (Group G rhizobia) immediately prior to planting.

Early vigor was estimated by measuring plant biomass and height at 625°d (45 days after sowing, assuming base temperature = 0°C). To estimate growth rates non-destructively, plant height was measured on 2 subsequent occasions (1023, 1389° d; 71, 95 days) during the linear growth phase. Phenological observations (onset and end of flowering, onset of podding, maturity) were made 3 times weekly from flowering onwards. At maturity (defined by 95% of pods being dry) all above-ground biomass was harvested, separated into vegetative and reproductive matter by branch order (mainstem, lateral and basal), and weighed after oven drying at 60°C for 48 h. Seed and pod numbers were counted and weighed within each branch order to facilitate the calculation of separate yield components (seed size, seeds per pod).

2007 growing season rainfall was low (331 mm), and the trial exposed to terminal drought, with little reproductive phase rainfall combined with relatively high temperatures and a high rate of temperature increase (Table 1).

### 2008 Field evaluation

To validate the 2007 results, a smaller, representative subset of wild and domesticated germplasm (*L. albus, n* = 17; *L. angustifolius, n* = 42; *L. luteus, n* = 29) was evaluated in a common garden in deep sands at the adjacent University of Western Australia field station (−31.95 N, 115.79 E) in 2008 using seed from the Australian Lupin Collection. Seeds were imbibed, vernalized at 4°C for 28 days prior to planting and hand planted in a RCBD (*n* = 4) on 19th June 2008 in single row plots (1.5 m long, 10 cm inter-plant, 20 cm inter-row distances). Phenological observations (flowering, end of flowering, maturity) were made 3 times weekly from flowering onwards. Biomass was destructively harvested in 1–3 plants per plot, and canopy height measured non-destructively in 5 adjacent plants per plot during the linear growth phase at 604, 905, 1450, and 1850°d (corresponding to 48, 72, 110 and 131 days).

While total growing season rainfall was lower in 2008 than 2007 (288 vs. 331 mm), reproductive phase rainfall was higher, considerably more frequent, and coincided with lower temperatures than in 2007 (Table 1).

### Loss of seed dormancy

To investigate the role of seed dormancy loss in adaptation to rainfall gradients, low and high rainfall subsets of wild *L. angustifolius (n* = 20) and *L. luteus (n* = 12) were selected. This rainfall contrast also has implications on summer temperatures. Both the summer maxima and diurnal temperature range tend to increase with aridity, rising with cluster number in these species (Table 1). Accordingly, the contrasting rainfall subsets of wild *L. angustifolius* and *L. luteus* investigated for loss of physical dormancy also represent contrasting summer temperatures (Table 2). Seed dormancy loss was not investigated in *L. albus* because in this species the distinction between wild material and escaped landraces is tenuous.

**Table 2:** **Average seasonal rainfall and summer temperatures (mean, maximum and diurnal range) in high and low rainfall subsets of wild *L. angustifolius* (n = 20) and *L. luteus (n* = 12) used in the investigation of hard seed breakdown over time**.

**Species/Category**	**Season rain**	**Summer temperatures (°C)**		
	**(mm)**	**Mean**	**Maximum**	**Diurnal range**
*L. angustifolius* L.	718	22.0	30.0	16.5
High rainfall	1022	20.7	28.2	15.5
Low rainfall	262	24.0	32.7	18.0
*L. luteus* L.	773	22.6	28.8	13.4
High rainfall	1064	19.7	25.7	12.7
Low rainfall	482	25.5	31.9	14.1

Seeds were multiplied in a common field at CSIRO Floreat (−31.95 N, 115.79 E) in 2014, harvested in November and immediately returned to the field to simulate Mediterranean climate hard seed breakdown in diurnally varying summer temperatures from December onwards according to the following protocol. 50 seeds per replication (RCBD, *n* = 4) were bagged in mesh pouches, and placed on the soil surface of the CSIRO rainout shelter amongst retained crop stubble on 18th December 2014. At approximately monthly intervals the seeds were allowed to imbibe water for 48 h, after which swollen and germinating seeds were recorded and removed. Remaining hard seeds were returned to the mesh pouches in the field and the process repeated to generate cumulative hard seed breakdown curves. Note that between December and May the rainout shelter was used to avoid uncontrolled seed wetting, whereas during the May-December growing season the pouches were exposed to rain as well as monthly imbibition for 48 h.

### Statistical analysis

The data was analyzed separately for each year using Genstat V16. Within and between species differences were investigated using ANOVA and linear regression, fitting species as main effects, clusters (Table 1) nested within species, and accessions nested within clusters. In all analyses, residual plots were generated to identify outliers, and confirm that variance was common and normally distributed. Transformations were made as appropriate. Principal components analysis (PCA) was used to describe the relationships between collection site bioclimatic data and within- and between-species biology using data in Berger et al. (2008) and ANOVA means from the 2007 and 2008 field experiments. PCA was based on a correlation matrix to remove scale effects between variables.

## Results

### Phenology and environment

**Figure 2:**
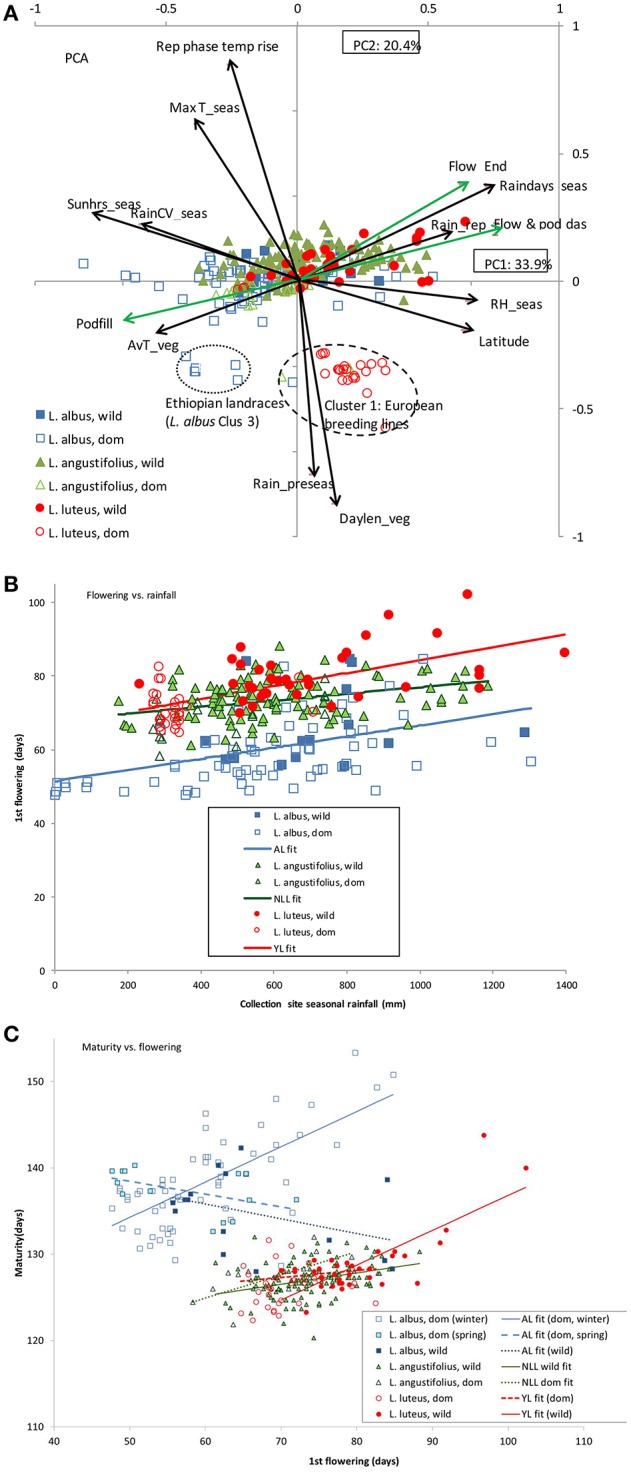
**Plant phenology and collection site environment in old word lupins. (A)** Principal components analysis of collection site bioclimatic variables and plant phenology data from a common garden 2007 field study of *L. angustifolius, L. luteus* and *L. albus*. Factor loadings for PC1 and 2 are presented as vectors (black for bioclimatic, green for biological variables), abbreviated as follows: av, average; CV, coefficient of variation; das, days after sowing; flow, flowering; pod, podding; podfill, pod filling phase; rain, rainfall; raindays, number of rainy days; RH, relative humidity; rep, reproductive phase; seas, season; sun hours; mean daily sunshine hours; T, temperature; veg, vegetative phase. Markers represent genotype scores, classified by species and domestication status. **(B)** Linear regression of flowering against collection site season rainfall, fitting species as factors, accounting for 55.8% of variance. Regression equations presented in Table 5. **(C)** Linear regression of maturity against flowering, fitting species and domestication status as factors, accounting for 79.5% of variance. Regression equations presented in Table 5.

To demonstrate the interaction between habitat climate and lupin phenology, PCA was performed on accession collection site bioclimatic and 2007 field phenology data in a single analysis. If phenology-climate matching is a key adaptation strategy in lupin, we expect to find vectors describing accession phenology and site climate aligned on the same dimension in the PCA. PC1 captured a drought stress gradient: seasonal sunshine hours, rainfall variability and vegetative phase temperature increasing; and latitude, seasonal relative humidity, rainfall frequency and reproductive phase rainfall decreasing from right to left (Figure 2A). Indeed, phenology and bioclimatic vectors were intermingled on PC1. Dates of flowering, podding and end of flowering increased, and pod fill decreased from left to right on PC1, in inverse proportion to the aforementioned drought stress gradient, particularly those vectors describing reproductive rainfall, rainfall frequency and average vegetative phase temperature. PC2 was dominated by negative loadings on vegetative phase daylength, pre-season rainfall, and positive loadings on maximum seasonal temperature and rates of reproductive phase temperature increase; separating the cooler domesticated European (all 3 species) and Ethiopian highland *L. albus* collection sites from their Mediterranean counterparts.

All 3 lupin species were broadly distributed along PC1 (particularly *L. albus*), indicative of a common, wide ranging stress gradient among collection sites, and a correspondingly common, wide phenological response. To investigate this directly, flowering date was regressed against collection site seasonal rainfall (Figure 2B). While flowering date was delayed as rainfall increased in all 3 species (*P* < 0.001), there were significant intercept and slope differences. *L. luteus* and *L. albus* were similarly responsive to collection site seasonal rainfall (16.4 days delay/1000 ml rainfall, P_diff_ = 0.538), but *L. luteus* flowered consistently 15 days later than *L. albus* across its rainfall range (Figure 2B, *P* < 0.001). *L. angustifolius* and *L. luteus* intercepts were similar (67 days, P_diff_ = 0.607), but *L. angustifolius* flowering was much less responsive (9 days delay/1000 ml rainfall, P_diff_ = 0.03), causing these species to diverge under high collection site rainfall (Table 3). Thus, in average Mediterranean climates, wild *L. albus* flowered very early (Cluster 5, 65 days), while *L. angustifolius* (Cluster 4, 73 days) was only marginally earlier than *L. luteus* (Cluster 3, 78 days). Conversely, in cool, high rainfall, long-season Iberian habitats, *L. albus* flowered in 70 days (Cluster 2), followed by *L. angustifolius* (Cluster 3, 78 days), while *L. luteus* was much later (Cluster 2, 89 days).

**Table 3:** **Within and between old world lupin species differences in terms of variance distribution^*a*^ and mean values for seed size, early growth, phenology and productivity in a rainfed field common garden evaluation in 2007 (See Table 4 for the 2008 common garden evaluation)**.

**Category**		**Seed size**	**Early vigor**	**Growth rate**	**Plant ht**	**Flower days**	**Pod days**	**Rep phase**	**Maturity**	**HI**	**Biomass**	**Seed no/pl**	**Seed wt/pl (log)**
^a^Species variance		98^***^	96^***^	75^***^	89^***^	92^***^	92^***^	98^***^	97^***^	94^***^	84^***^	92^***^	92^***^
^a^Cluster within species v.		2^***^	3^***^	19^***^	7^***^	7^***^	7^***^	2^***^	2^***^	4^***^	10^***^	6^***^	6^***^
^a^Accession within cluster		0.2^***^	1^***^	6^***^	3^***^	1^***^	1^***^	0.3^***^	1^***^	2^***^	5^***^	2^***^	2^***^
Species means	*n*												
*L. albus* L.	84	26.8	0.68	0.93	57.8	61	73	64	138	38.4	69.8	107.4	1.3
*L. angustifolius* L.	127	10.1	0.29	0.80	48.5	73	81	46	127	34.8	56.9	201.9	1.2
*L. luteus* L.	69	11.9	0.34	0.86	50.6	76	84	43	128	30.4	53.5	137.8	1.1
LSD Species mean		1.2	0.05	0.08	1.3	1	1	1	1.2	3.0	9.7	15.2	0.1
Cluster means	***L. albus*** **L**.												
Albus 1, Br	1	21.5	0.26	0.41	32.3	56	67	67	129	30.7	16.3	23	0.6
Albus 2, LR	6	27.8	0.83	0.98	60.1	70	82	62	143	33.1	66.4	81	1.3
Albus 3, LR	2	23.5	0.71	1.06	64.6	67	77	62	143	46.1	61.0	119	1.3
Albus 3, W	3	18.6	0.50	0.74	45.3	72	82	50	133	41.3	45.4	93	1.2
Albus 4, LR	6	21.1	0.50	0.79	50.6	65	80	63	144	33.3	44.8	72	1.1
Albus 5, LR	42	30.2	0.77	0.98	60.9	59	71	66	137	39.0	75.4	103	1.4
Albus 5, W	12	23.8	0.52	0.88	52.8	65	76	59	135	37.4	64.4	109	1.3
Albus 6, LR	14	24.6	0.62	0.94	57.9	57	71	67	137	39.2	74.4	134	1.4
Albus 7, LR	8	30.1	0.75	0.92	61.2	52	65	70	135	45.7	84.4	140	1.5
LSD *albus*		2.8	0.25	0.25	9.0	4	4	5	4	10.0	29.3	57	0.3
***L. angustifolius*** **L**.													
Angus 1, Br	1	14.5	0.43	0.80	50.5	74	80	47	127	40.2	36.0	101	1.2
Angus 2, W	17	7.3	0.24	0.91	52.1	80	87	41	128	30.6	47.7	213	1.0
Angus 3, W	11	8.2	0.25	0.88	51.0	78	85	42	127	34.6	52.1	218	1.1
Angus 4, Br	15	12.3	0.34	0.73	48.0	68	76	51	127	32.7	48.1	130	1.1
Angus 4, W	76	10.3	0.28	0.79	47.7	73	80	47	127	35.9	58.5	208	1.2
Angus 5, W	7	11.3	0.43	0.72	44.6	69	78	52	127	36.8	63.9	250	1.3
LSD *angustifolius*		2.1	0.13	0.23	8.5	4	4	5	3	9.9	34.9	130	0.2
***L. luteus*** **L**.													
Luteus 1, Br	28	12.7	0.39	0.85	51.5	71	80	46	127	33.3	46.5	121	1.0
Luteus 2, W	7	8.6	0.26	0.80	43.8	89	96	37	133	22.3	51.2	130	0.9
Luteus 3, W	29	11.8	0.31	0.90	51.6	78	86	41	127	28.9	63.3	161	1.2
Luteus 3, Br	5	12.7	0.38	0.82	49.6	71	80	47	128	33.1	39.6	114	1.1
LSD *luteus*		1.1	0.07	0.18	4.6	2	2	2	2	5.0	17.2	48	0.2

a Species, Cluster within species, Accession within cluster. Percentage of variance captured by each classification level in nested ANOVA.

*** *P < 0.001*.

While flowering and maturity date tended to be positively correlated, there were again considerable differences within and between species (Figure 2C, Table 3). *L. albus* tended to mature much later than the other species. While, domesticated, winter-sown *L. albus* had a very late, strongly flowering-responsive maturity, its spring-sown and wild counterparts did not delay maturity as flowering date increased (Figure 2C). *L. angustifolius* matured earliest (Table 3), was less flowering-responsive than the remaining species, and there were no differences between domesticated and wild material (Figure 2C, P_diff_ = 0.278), nor between clusters (Table 3). Domesticated *L. luteus* was similar to *L. angustifolius*, while wild material was as flowering-responsive as domesticated, winter-sown *L. albus* (Figure 2C), driven by late flowering and maturity in Cluster 2 (Table 3).

### Phenology, growth, and productivity within and between species

**Figure 3:**
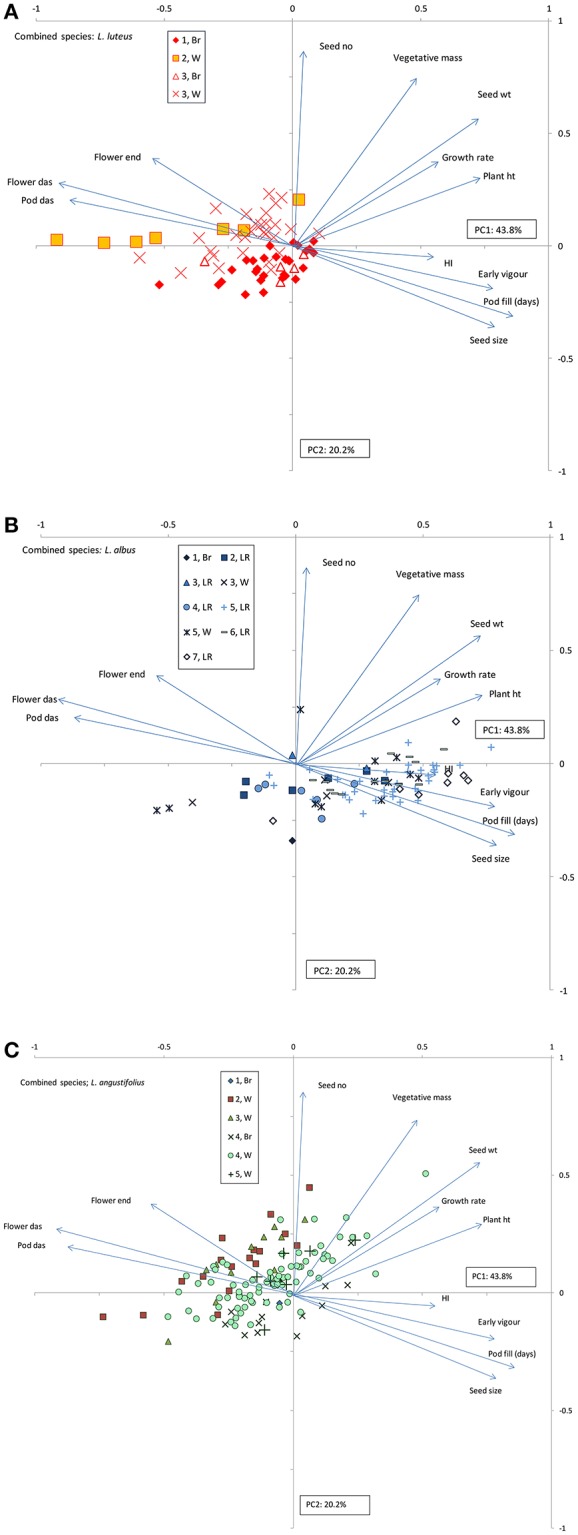
**Combined species principal components analysis (PCA) of old world lupin phenology, growth and productivity under rainfed field conditions in 2007**. *L. luteus* **(A)**, *L. albus* **(B)**, and *L. angustifolius* **(C)** are plotted in 3 separate panels with identical vectors to clarify within species cluster differences. Factor loadings for PC1 and 2 are presented as vectors, abbreviated as follows: das, days after sowing; HI, harvest index; ht, height; no, number; wt, weight. Markers represent genotype scores, classified by habitat/domestication status clusters within species.

To highlight the effect of this phenological variability on growth and productivity, a combined species PCA was performed, accounting for 64% of variance in 2 components (Figure 3). PC1 captured a phenology-productivity continuum with negative loadings on flowering and podding, and positive loadings on seed size, early vigor, length of the reproductive phase (pod fill), harvest index, plant growth rates, height and productivity. PC2 was dominated by positive loadings on seed number and vegetative biomass.

Despite considerable within-species variation, the 3 old world lupin species plotted discretely on Figure 3, reflecting considerable between species variance for all traits in the 2007 field evaluation (Table 3). *L. luteus* was located on the left of Figure 3A, indicative of late phenology, small seed size, low harvest index, growth rates and productivity (Table 3). Within *L. luteus* there were clear cluster differences in both PC1 and 2 (Figure 3A). Wild germplasm, particularly Cluster 3, tended to have more positive PC2 scores than domesticated material, associated with higher seed numbers per plant (Table 3). Domesticated PC1 scores were more positive than wild, reflecting strong selection for early phenology, high early vigor, harvest index and larger seed size (Table 3: Cluster 1, Cluster 3, Br vs W). A similar contrast occurred within wild *L. luteus:* with higher PC1 scores, earlier flowering, higher vigor, and productivity in low than high rainfall ecotypes (Table 3: Clusters 3, W vs. 2, W).

Despite a wide PC1 range, most *L. albus* accessions were located in the lower-right quadrant of Figure 3B, characterized by early phenology, large seeds, high early vigor, rapid growth rates, and high plant height, harvest index, seed and biological yield (Table 3). PC2 scores were predominantly negative in *L. albus*, indicative of low seed number. There were pronounced cluster differences along the phenology-productivity continuum in PC1, from the very early warm season, southern and spring-sown Mediterranean landraces (Clusters 7, 6) on the far right, through to the much later cool climate Iberian and Mediterranean germplasm (Clusters 2, 3) on the left. Cluster 5, representing average Mediterranean climates, was characterized by very wide ranging PC1 scores, exacerbated by the inclusion of 2 very late wild Greek outliers on the far left of PC1. Ethiopian *L. albus* (Cluster 4) formed a tightly clustered group in the middle of the phenology-productivity continuum. Wild-domesticated comparisons were feasible in Clusters 3 and 5. In both cases, PC1 scores were higher in domesticated than wild material, indicating similar selection for early phenology, high early vigor, harvest index and larger seed size as in *L. luteus* (Table 3).

*Lupinus angustifolius* was intermediate between *L. luteus* and *L. albus* along the PC1 phenology-productivity continuum, while PC2 scores tended to be more positive (Figure 3C), reflecting its greater fecundity (Table 3). Unlike the other 2 lupin species, *L. angustifolius* clusters were better aligned along a phenology-seed size/early vigor continuum described by the vectors mapping to the upper left and lower right quadrants of Figure 3C. Among the wild material, the cool climate Mediterranean and Iberian germplasm (Clusters 2, 3) was largely distributed along the upper left of Figure 3C, indicative of late phenology, small seed size and low early vigor (Table 3). Conversely, low rainfall North African material (Cluster 5) was located on the lower right, and characterized by early flowering, greater early vigor, and larger seed. Average Mediterranean climate (Cluster 4) germplasm was intermediate between these groups and very widely distributed, similar to its *L. albus* counterparts. Domesticated *L. angustifolius* was located on the lower-right extreme of Figure 3C, reflecting selection for earliness, high early vigor and large seed size (Table 3; Cluster 4, Br vs W). All *L. angustifolius* clusters ranged widely along a 45° axis defined by the lower left and upper right quadrants of Figure 3C, indicative of considerable within-cluster variation for plant height, growth rate and productivity.

**Figure 4:**
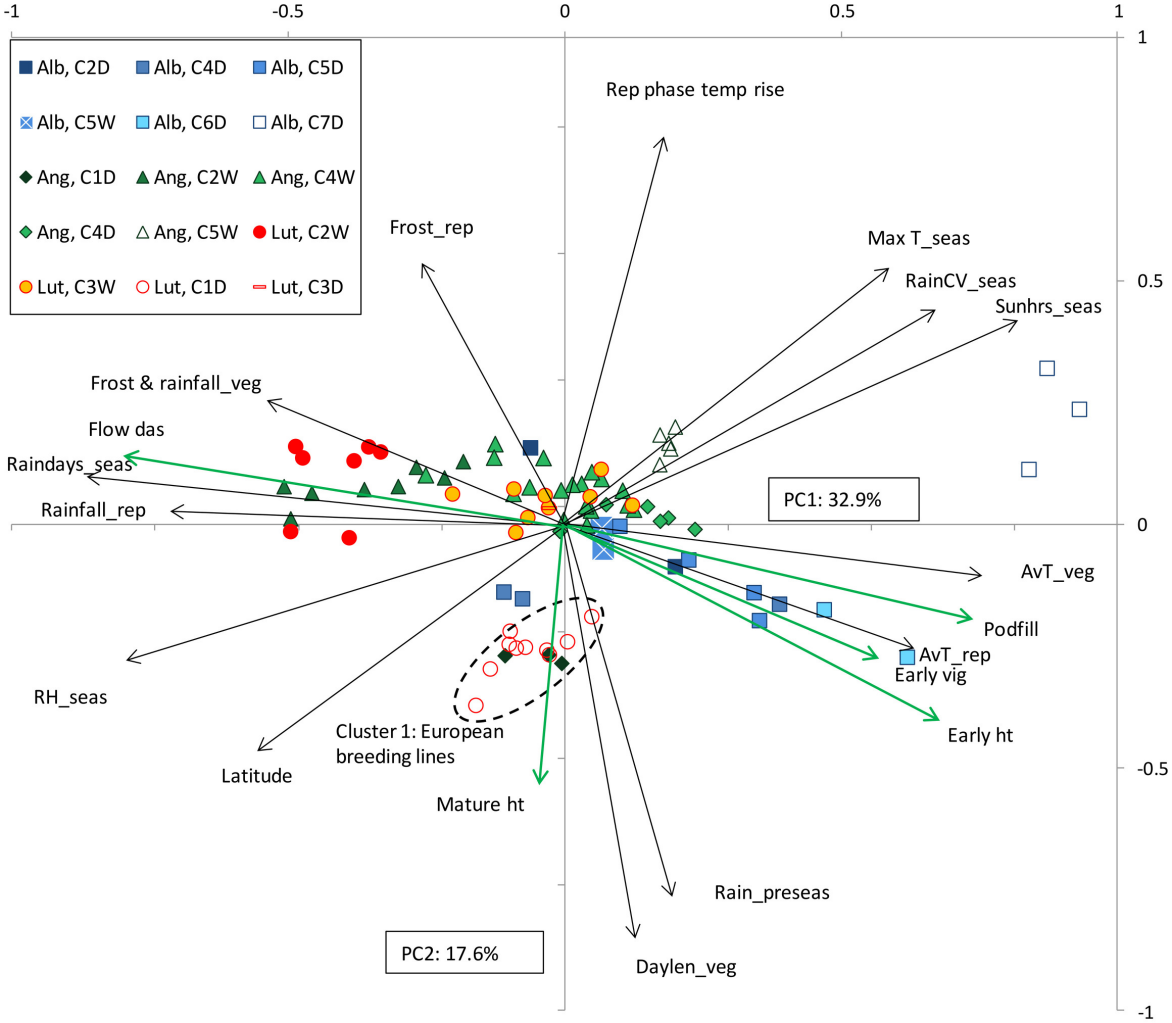
**Combined species principal components analysis (PCA) of old world lupin (*L*. *angustifolius*, Ang; *L. luteus*, Lut; and *L. albus*, Alb) collection site climate, phenology, growth and vigor data from a common garden field study in 2008**. Factor loadings for PC1 and 2 are presented as vectors (black for bioclimatic, green for biological variables), abbreviated as in Figures 2, 3.

Combined species PCA was repeated on the 2008 data with remarkably similar results, returning clear between and within species cluster separation along a phenology/vigor-drought stress continuum on PC1, and separating Mediterranean from domesticated European and Ethiopian material along PC2 (Figure 4; see Table 4 for species and cluster means). As in 2007, balanced within-cluster comparisons confirmed higher vigor/earlier phenology in domesticated compared to wild material in the 2008 data (Table 4, Figure 4: *L*. *angustifolius*, Cluster 4, and *L. albus*, Cluster 5).

**Table 4:** **Within and between old world lupin species differences in terms of variance distribution^a^ and mean values for seed size, early growth, phenology and productivity in a rainfed field common garden evaluation in 2008**.

**Category**		**Early vigour**	**Growth rate bio**	**Growth rate ht**	**Plant ht**	**Flower days**	**Rep phase**	**Maturity days**	**Biomass**
^a^Species variance		93.6^***^	54.4^***^	64.2^***^	58.2^***^	90.7^***^	96.7^***^	95.1^***^	56.1
^a^Cluster within species		4.4^***^	24.7^***^	28.5^***^	29.5^***^	8.2^***^	2.4^***^	3.3^***^	24.8
^a^Accession within cluster		2.0^***^	20.9^***^	7.4^***^	12.3^***^	1.2^***^	0.9^***^	1.6^***^	19.2
Species means	*n*								
*L. albus* L.	17	0.65	0.023	0.041	56	61	103	164	27.9
*L. angustifolius* L.	41	0.32	0.019	0.035	50	71	81	152	22.5
*L. luteus* L.	28	0.38	0.018	0.038	50	82	72	153	22.7
LSD Species mean		0.03	0.003	0.004	1.8	0.7	1.4	1.3	2.5
Cluster means	***L. albus*** **L**.								
Albus 2, LR	2	0.67	0.026	0.046	62	64	104	167	33.5
Albus 4, LR	3	0.50	0.018	0.040	56	66	103	169	22.7
Albus 5, LR	4	0.71	0.024	0.044	62	60	106	166	28.9
Albus 5, W	2	0.64	0.025	0.046	54	68	91	160	32.2
Albus 6, LR	3	0.57	0.026	0.041	57	61	102	165	35.2
Albus 7, LR	3	0.77	0.016	0.028	43	51	107	158	17.5
LSD *albus*		0.14	0.010	0.012	6.5	2.1	3.3	3.3	9.9
***L. angustifolius*** **l**.									
Angus 1, Br	3	0.38	0.022	0.041	58	71	81	152	25.0
Angus 2, W	8	0.24	0.017	0.042	54	77	77	154	20.3
Angus 3, W	2	0.17	0.016	0.033	42	76	75	152	17.6
Angus 4, Br	6	0.38	0.022	0.033	51	66	88	153	25.5
Angus 4, W	18	0.35	0.020	0.034	48	70	81	151	23.8
Angus 5, W	5	0.26	0.014	0.029	42	68	84	153	17.8
LSD *angustifolius*		0.09	0.008	0.010	6.3	2.4	5.9	5.0	8.7
***L. luteus*** **L**.									
Luteus 1, Br	10	0.41	0.017	0.036	51	75	76	151	21.8
Luteus 2, W	7	0.27	0.019	0.041	49	95	62	156	22.1
Luteus 3, Br	1	0.36	0.013	0.038	47	79	74	153	13.4
Luteus 3, W	10	0.43	0.020	0.038	49	80	73	153	24.9
LSD *luteus*		0.12	0.009	0.011	5.9	2.7	4.7	4.2	10.5

a Species, Cluster within species, Accession within cluster. Percentage of variance captured by each classification level in nested ANOVA.

*** *P < 0.001*.

**Table 5:** **Linear equations for relationships between flowering, maturity, seed size and pod fill in Old World lupin groups regressed in Figures 2B,C, 5B,C**.

**Species**	**Flowering-rainfall (Figure** **2B****)**		**Maturity-flowering (Figure** **2C****)**		**Flower-seed size (Figure** **5B****)**		**Pod fill-flower (Figure** **5C****)**	
	**Intercept**	**Slope**	**Intercept**	**Slope**	**Intercept**	**Slope**	**Intercept**	**Slope**
*L. albus*, dom (winter)	51.4^***^	0.015^***^	113.8^***^	0.41^***^	40.4^***^	−0.18^**^	101.4^***^	−0.60^***^
*L. albus*, dom (spring)	51.4^***^	0.015^***^	145.9^***^	−0.15^(= 0.09)^	−1.5^NS^	0.46^***^	132.3^***^	−1.15^***^
*L. albus*, wild	51.4^***^	0.015^***^	146.1^***^	−0.17^*^	59.1^***^	−0.55^***^	124.7^***^	−1.02^***^
*L. angustifolius*, dom	68.0^***^	0.009^***^	108.9^***^	0.27^*^	15.4^NS^	−0.04^NS^	87.2^***^	−0.53^***^
*L. angustifolius*, wild	68.0^***^	0.009^***^	117.3^***^	0.13^**^	30.3^***^	−0.28^***^	104.3^***^	−0.79^***^
*L. luteus*, dom	66.7^***^	0.18^***^	120.6^***^	0.08^NS^	15.4^NS^	−0.04^NS^	96.8^***^	−0.71^***^
*L. luteus*, wild	66.7^***^	0.18^***^	96.4^***^	0.40^***^	31.3^***^	−0.25^**^	81.1^***^	−0.50^***^
LSD (*P* < 0.05)	3.4	0.005	10.4	0.15	17.5	0.26	11.0	0.16

P-values present outcomes from T-tests of H_0_ intercept and slope = 0:

NS > 0.05,

* < 0.05,

** < 0.01,

*** *< 0.001*.

**Figure 5:**
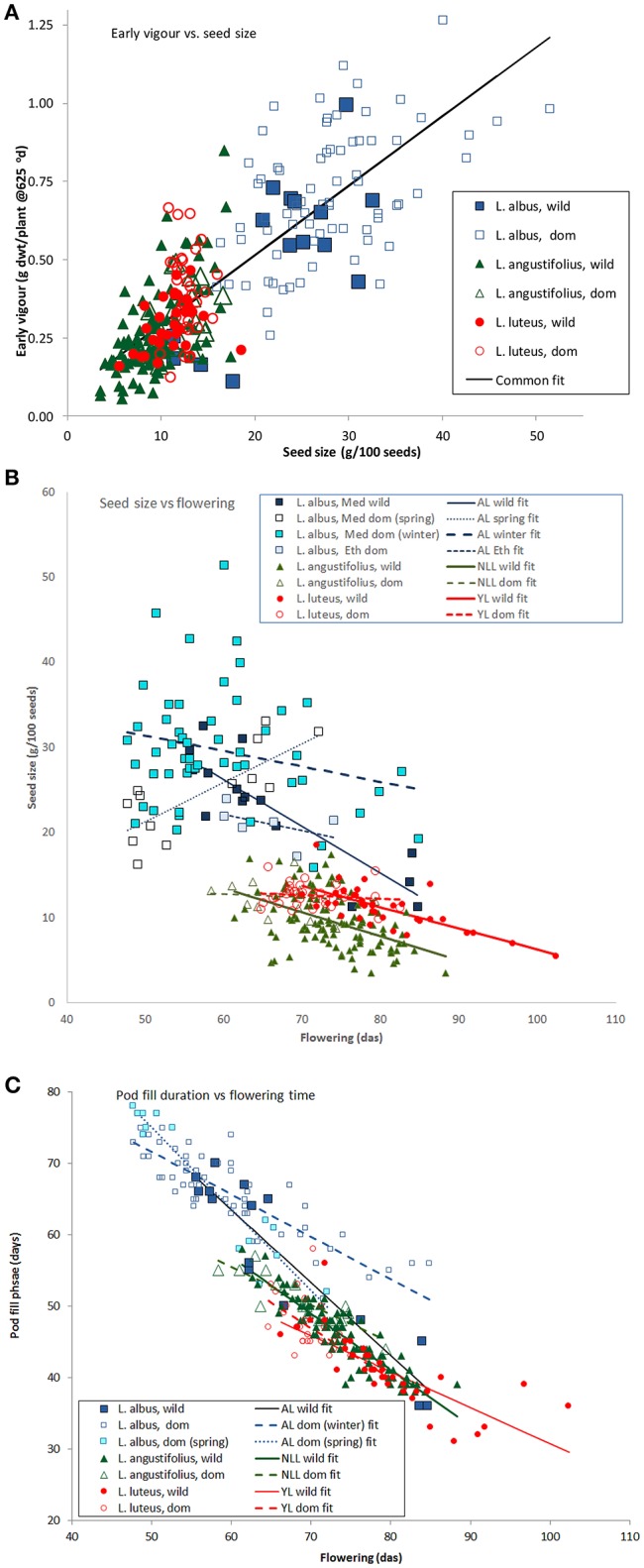
**The effects of seed size on early vigor (A)**, and flowering date on seed size **(B)**, and the length of the reproductive phase **(C)** in old world lupins (data from a 2007 common garden field trial). In **(A)** a common linear regression accounted for 66.2% of variance, while in **(B,C)** separate regression equations for species accounted for 82.5 and 93.1%, respectively. (Regression equations for **(B,C)** presented in Table 5).

Regression was used to more closely investigate the consistent phenology trade-offs highlighted in PC1 in both 2007 and 2008 ordinations. Early vigor increased in proportion to seed size in a common manner among all 3 species (Figure 5A: *y* = 0.07 + 0.02x, *P* < 0.001). Seed size was negatively related to flowering date, largely decreasing at similar rates between or within species as flowering became later (Figure 5B, Table 3). Domesticated *L. luteus* and *L. angustifolius* were notable exceptions where there was no significant relationship between seed size and flowering date (Table 3). Wild *L. albus* appeared to be more responsive than the others, while in domesticated, spring-sown *L. albus*, seed size appeared to increase, rather than decrease with flowering time (Figure 5B, Table 3). Note that both these groups were relatively small, and regressions subject to leverage effects. The length of the reproductive phase was also negatively related to flowering date across all species, with the strongest responses in wild and domesticated spring-sown *L. albus* (Figure 5C, Table 3). While winter-sown *L. albus* and *L. luteus* responses were parallel, *L. albus* pod fill phase was consistently longer throughout its flowering range than *L. luteus* (Figure 5C, Table 3), reflecting its later maturity date.

**Figure 6:**
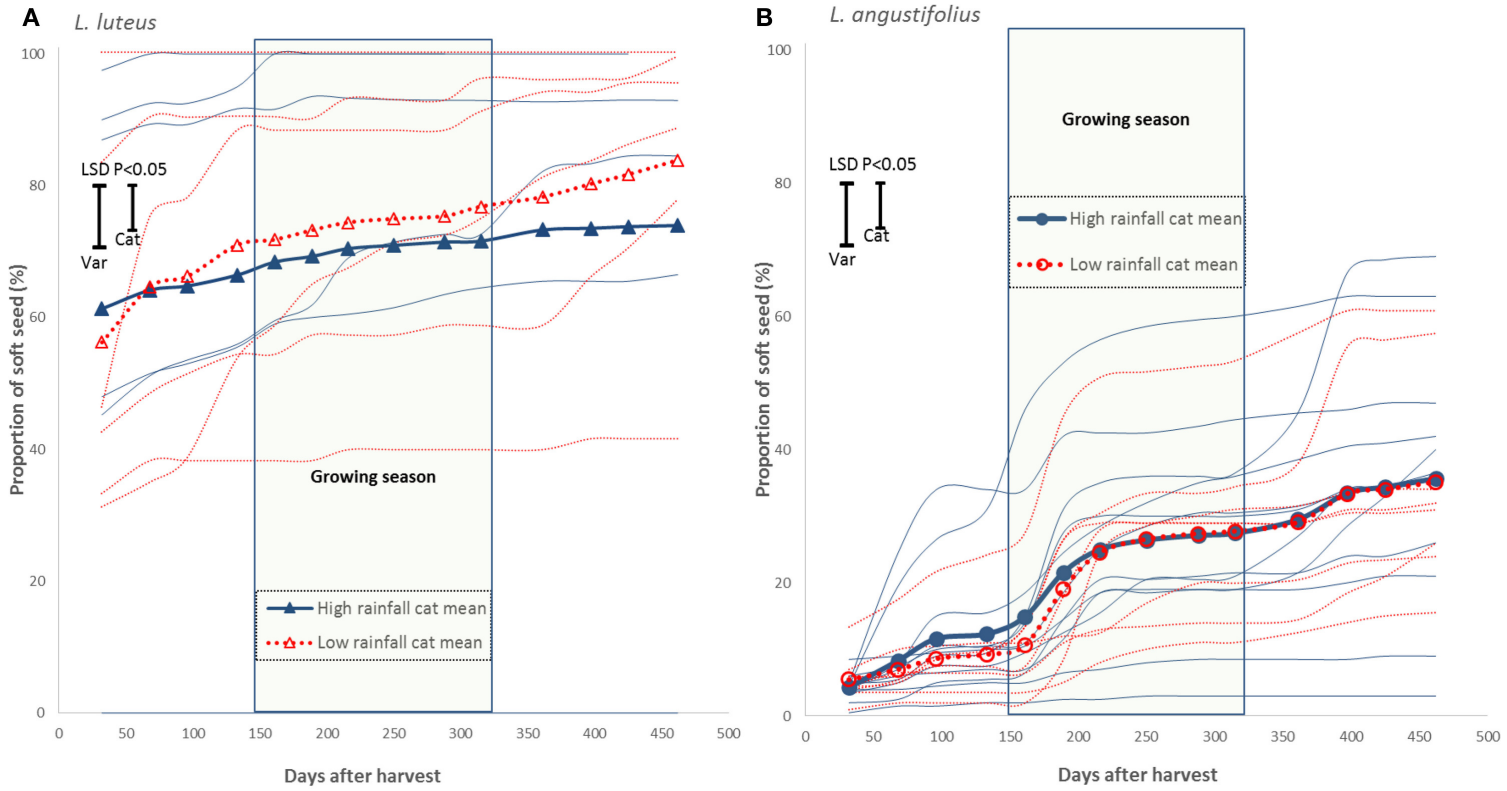
**Hard seed breakdown in wild, low (…) and high rainfall (—) *L. luteus* (A, Δ)**, and *L. angustifolius* **(B, ◦)**. Genotype responses plotted in narrow curves without markers, category mean responses plotted in wide, bolded curves with markers for each sample point.

Hard seed breakdown patterns were typically logistic in both *L. luteus* and *L. angustifolius* (Figure 6). Unlike the data presented previously, both species showed considerable genotypic variation that was unrelated to rainfall, such that there were no significant differences in both the starting intercepts and rates of hard seed breakdown between low and high rainfall ecotypes. Thus, during the first growing season after seed maturation high rainfall ecotypes could be extremely soft, or completely impermeable to water, and a similar range existed within low rainfall ecotypes (Figure 6). This situation appeared to continue into the 2nd year, notwithstanding further loss of physical dormancy in the second summer. Nor were there any relationships between early season soft seed proportion and flowering time or summer temperature mean, maxima or range in either species (*L. luteus, r*^2^ = 0.02–0.13, *P* = 0.494–0.959; *L. angustifolius, r*^2^ = 0.03–0.08, *P* = 0.123–0.579, data not presented). Nevertheless, there were clear cut specific differences: the proportion of soft seed tended to be far higher in *L. luteus* than in *L. angustifolius*. Seed banks of almost all *L. luteus* accessions were >30% soft at harvest, rising >70% during the first growing season. By contrast, the *L. angustifolius s*eed bank was typically <10% soft at harvest, rising to <30% during the first growing season, well after the onset of opening rains (Figure 6).

## Discussion

Our work demonstrates strong trade-offs between seed size, early vigor and phenology among and within Old World lupin species. Early vigor increases with seed size, while flowering becomes earlier as seed size increases, suggesting that phenology and seed size are complementary among the large-seeded Old World lupins, as observed in much smaller seeded Mediterranean pasture legumes (Cocks, 1999). In the Mediterranean climate of our common garden experiments, flowering time and the length of the pod filling phase were strongly negatively correlated: late flowering plants had short reproductive phases, ended by the ubiquitous terminal drought. Although the Old World lupin species occupy opposing corners of these trades-off, from early flowering, large-seeded, high vigor *L. albus* through *L. angustifolius* to its polar opposite: late, small-seeded, low vigor *L. luteus;* natural and human selection have operated in very similar ways in all 3 species. Among the wild material, as collection environments become more prone to terminal drought, phenology becomes earlier in all species, while seed size, early vigor and reproductive investment increase. Wild and domesticated germplasm separate along similar lines. Comparisons within similar habitat types demonstrate that domesticated material is consistently earlier flowering in all 3 species, and has larger seeds, greater early vigor and higher reproductive investment than wild, regardless of whether domestication was ancient or modern.

The role of phenology in avoiding perennial stresses, such as winter cold and terminal drought is well understood in Mediterranean annuals (Berger et al., 2016), and can lead to the evolution of regionally appropriate control mechanisms (Berger et al., 2011). This lupin example suggests that early phenology may also be important in supporting the production of larger seeds. In a growing season terminated by drought, early flowering allows for long pod filling phases (Figure 5C) that support the production of large seeds, important because these are likely to require more time to fill (Vile et al., 2006). Moreover, a long reproductive phase will reduce fecundity-seed size trade-offs in large seeded genotypes (Norman et al., 2005) by providing more opportunities for seed production. The reverse argument applies equally well to late flowering, high rainfall ecotypes. Here smaller, presumably faster developing seeds (Vile et al., 2006) reduce the fecundity constraints of a shorter reproductive phase.

So why should drought-prone Mediterranean habitats select for larger seeds? Negative correlations between seed size and moisture availability occur across wide-ranging community types in Californian herbs (Baker, 1972), while Mazer (1989) found that early and short duration flowering were associated with the production of large seeds in Indiana dune angiosperms. Similarly, Norman et al. (2005) found negative correlations between seed size and flowering date among 19 *Trifolium* species sourced from Mediterranean climates in the eastern Mediterranean and southern Australia. The role of seed size in promoting early establishment, vigor and competition in annual species is well known (Leishman et al., 2000). Therefore, under unfavorable conditions (such as aridity or shading) seed size is positively correlated to reproductive yield, trumping the usual fecundity-size trade-off (Venable and Brown, 1988). Rainfall amount and variability are inversely related cross the Mediterranean lupin range: drier sites tend to have more variable, less frequent rainfall (Berger et al., 2008, see also vectors in Figures 2, 4). Accordingly, it is important for low rainfall ecotypes to establish as quickly as possible after the growing season opening rains because the timing of the next rainfall is uncertain. This issue is exacerbated in Old World lupins, which are adapted to poorly fertile, acid sandy soils with little water-holding capacity, where the wetting front is likely to move rapidly downwards (Palta et al., 2012). Under these circumstances, maximizing early vigor in low rainfall ecotypes seems appropriate in the Old World lupins.

Given the potential for the regulation of germination to frame in-season phenology, and expected trade-offs between seed size and dormancy (Venable and Brown, 1988), it was interesting not to find a relationship between hardseed breakdown and collection site aridity in the “Old World” lupins (Figure 6). Low rainfall collection sites tend to have higher summer temperature maxima and wider diurnal ranges than high rainfall sites (Table 2). While there is clearly a strong summer pattern of hardseed breakdown, variation in intercepts and rates of dormancy loss suggest that this process has elements of both predictive and bet-hedging strategies (Donaldson-Matasci et al., 2013). The former protects against untimely germination, while the latter spreads risk over time. Both responses vary widely between *L. luteus* and *L. angustifolius* low and high rainfall ecotypes such that these populations are buffered by a range of germination strategies. Even in strongly Mediterranean climates rainfall variability may be too high to select for a uniform predictive dormancy response, especially given low water holding capacity soils, and the likelihood of other unpredictable disturbance, such as grazing. Moreover, there are other advantages attributed to diversified bet-hedging related to the effective reduction in seed density associated with staggered germination. These include escaping crowding, reducing sib competition and the potential for inbreeding (Rubio de Casas et al., 2015). Indeed, studies of Mediterranean legumes often return very weak (Piano et al., 1996) or no relationships between hardseed breakdown and flowering time or collection site rainfall (Gladstones, 1967; Norman et al., 2002).

The overlap between natural and human selection in all 3 species is particularly interesting. Domesticated preferences have consistently selected for the “drought-adapted package” even though domestication occurred in very different environments, separated by 1000s of years. *L. albus* was domesticated in the Aegean during the Bronze Age (Zohary and Hopf, 2000); while *L. luteus* and *L. angustifolius* were domesticated as spring-sown temperate European crops in the 18–19th centuries (Hondelmann, 1984), moving to Mediterranean Australia in the late twentieth century (Gladstones, 1994). *L. albus* is traditionally grown for human consumption as a whole seed where larger seeds are preferred (Huyghe, 1997), therefore seed size likely to have been under direct selection. Moreover, as a Mediterranean crop planted after the season opening rains (or indeed considerably later, as in the case of Balkan spring-sown crops; Mihailovic et al., 2008) its development is delayed compared to those hard-seeded wild populations germinating on soil moisture, exerting strong selection pressure for early vigor/phenology to catch up and complete the lifecycle prior to the onset of terminal drought. The European domestications selected for similar trait combinations, but for different reasons. Early attempts to domesticate *L. albus* failed due to the inability of the crop to mature in the absence of terminal drought (Hondelmann, 1984). Subsequent efforts with *L. luteus* and *L. angustifolius* selected strongly on phenology and early vigor, attested to by cultivar names such as Pflugs Allerfrühste (plow's earliest) (Gladstones, 1970), and reflected in the early, flowering-unresponsive maturity of domesticated material in this study. Later Australian efforts in warm, short season Mediterranean environments greatly enhanced this prior selection for earliness, selecting for extremely temperature responsive phenology (Berger et al., 2012a,b), equivalent to that of Southern Indian chickpea, an environment <10°C warmer than the northern Western Australian grainbelt (25.8° vs. 14.5°C) (Berger et al., 2011).

Having discussed similarities among the 3 Old World lupin species, it is important to consider the differences. While all species delay flowering with increasing collection site rainfall, *L. luteus* is particularly responsive, and comes from a late flowering baseline (intercept). Even low rainfall ecotypes (Cluster 3) are relatively late, a phenology that is delayed even more in high rainfall environments such as northern Iberia (Figure 2B). This is complemented by a greater tendency for soft seededness, such that a large proportion will germinate very early, perhaps allowing late flowering *L. luteus* to transition to reproduction at the same time as the much harder seeded *L. angustifolius*. In high rainfall ecotypes of *L. luteus*, the combination of early germination and late phenology drives a very long vegetative phase. This underpins massive above and below-ground biomass development, driving very high rates of water-use compared to earlier flowering, lower biomass low rainfall ecotypes (Berger and Ludwig, 2014). We assume that this aggressive strategy increases the competitive capacity of high rainfall *L. luteus* ecotypes, as high root biomass facilitates exploitation of nutrients and water, while large leaf area provides the C fixation to drive prolonged high growth rates, both of which are complemented by early establishment (Gremer et al., 2016). However, high water use leads to early stress onset under terminal drought. This is partly mitigated in *high* rainfall ecotypes only, by the generation of lower critical leaf water potentials and maintenance of higher relative water contents (Berger and Ludwig, 2014). Clearly there are limits to this drought tolerance capacity, as evidenced by low productivity of high rainfall ecotypes in the terminal drought stressed 2007 common garden. Accordingly, the early emergence, long vegetative phase, high biomass, resource acquisition and water-use lifecycle of *L. luteus* is a high risk, competitive strategy for specific adaptation to longer season, high rainfall environments, which may explain the scarcity of the species in the more drought-prone southern Mediterranean (Figure 1). Certainly, *L. luteus* has the narrowest, most coastal distribution of the 3 Old world species in the present study (Gladstones, 1998). Yellow lupin has struggled to establish itself as a break crop in Mediterranean agro-ecosystems, and our results suggest that re-imagining its role in the system is warranted. Perhaps there is a case for an extremely early-sown, drought tolerant, late variety for Mediterranean high rainfall zones?

Conversely, *L. angustifolius* is widely distributed around the Mediterranean (Gladstones, 1998). High rainfall ecotypes appear to be much more conservative than in *L. luteus*, with a higher proportion of hard seed, flowering and maturing much earlier, with a relatively rainfall-unresponsive phenology (Figures 2B,C). These characteristics identify *L. angustifolius* as a typical drought escaping species (Berger et al., 2016), and therefore we wonder to what extent there are adaptive traits that can be exploited for higher rainfall environments, such as the competitive, but risky, high productivity strategy of *L. luteus*. Currently, there are no elite narrow leafed lupin cultivars targeting long season, high yield potential environments (Berger et al., 2012a; Chen et al., 2016).

*Lupinus albus* is very different: flowering earlier but maturing much later than *L. luteus* and *L. angustifolius*; leading to longer reproductive phases that support much larger seed sizes and early vigor. This ruderal/competitive combination appears to give *L. albus* a very broad adaptive capacity, reflected in its relatively wider Mediterranean and North African distribution (Figure 1; Gladstones, 1998). How *L. albus* combines early flowering and late maturity is unknown. Perhaps its greater early vigor allows the species to access more water from lower in the soil profile than *L. luteus* and *L. angustifolius*, sustaining a longer reproductive phase under terminal drought. *L. albus* seedlings certainly accumulate much greater lateral root numbers, total root length and biomass, at higher length to weight ratios than either *L. luteus* or *L. angustifolius* (Clements et al., 1993). Alternatively, the species may be less profligate than the others in its vegetative water-use, saving water for the reproductive phase. This strategy has been documented in crops adapted to fine-textured, higher water-holding capacity soils, such as chickpea and pearl millet (Kholová et al., 2010; Zaman-Allah et al., 2011), but is harder to credit in such a vigorous, coarse-textured soil adapted species.

We conclude that while trade-offs between phenology, seed size and vigor operate in a similar manner in these 3 Old World lupin species as they transition from low to high rainfall environments, or from wild Mediterranean annual to domesticated crop, the combination of these and associated traits defines their adaptive potential, and is reflected in their natural distribution. Given that *L. luteus, L. angustifolius* and even *L. albus* are still relatively unimproved, partly domesticated crops, plant breeders can exploit these relationships to increase yield in target environments.

## Author contributions

JB designed helped implement the experiments, analyzed the results, and wrote the paper. DS, CL implemented the experiments, and provided feedback on the paper.

### Conflict of interest statement

The authors declare that the research was conducted in the absence of any commercial or financial relationships that could be construed as a potential conflict of interest.
